# Scaling of Brain Metabolism with a Fixed Energy Budget per Neuron: Implications for Neuronal Activity, Plasticity and Evolution

**DOI:** 10.1371/journal.pone.0017514

**Published:** 2011-03-01

**Authors:** Suzana Herculano-Houzel

**Affiliations:** 1 Instituto de Ciências Biomédicas, Universidade Federal do Rio de Janeiro, Rio de Janeiro, Rio de Janeiro, Brasil; 2 Instituto Nacional de Neurociência Translacional, Ministério de Ciência e Tecnologia, São Paulo, São Paulo, Brasil; University of Maribor, Slovenia

## Abstract

It is usually considered that larger brains have larger neurons, which consume more energy individually, and are therefore accompanied by a larger number of glial cells per neuron. These notions, however, have never been tested. Based on glucose and oxygen metabolic rates in awake animals and their recently determined numbers of neurons, here I show that, contrary to the expected, the estimated glucose use per neuron is remarkably constant, varying only by 40% across the six species of rodents and primates (including humans). The estimated average glucose use per neuron does not correlate with neuronal density in any structure. This suggests that the energy budget of the whole brain per neuron is fixed across species and brain sizes, such that total glucose use by the brain as a whole, by the cerebral cortex and also by the cerebellum alone are linear functions of the number of neurons in the structures across the species (although the average glucose consumption per neuron is at least 10× higher in the cerebral cortex than in the cerebellum). These results indicate that the apparently remarkable use in humans of 20% of the whole body energy budget by a brain that represents only 2% of body mass is explained simply by its large number of neurons. Because synaptic activity is considered the major determinant of metabolic cost, a conserved energy budget per neuron has several profound implications for synaptic homeostasis and the regulation of firing rates, synaptic plasticity, brain imaging, pathologies, and for brain scaling in evolution.

## Introduction

The scaling of brain metabolism has important implications for brain function and evolution. The brain is the third most energy-expensive organ in the human body, ranking in total organ metabolic cost below skeletal muscle and liver only [1]. While the metabolic needs of most body organs are closely associated with body size, such that the relative metabolic cost of an organ depends on its relative size [2], the relative metabolic needs of mammalian brains are variable: excluding humans, the relative cost of the vertebrate brain ranges between 2 and 10% of the whole body metabolic cost [3]. This is attributable in part to the large variation in relative brain size across species, and in part to the constantly high metabolic activity of the brain, regardless of the behavioral state of the animal [4]. In contrast, the human brain, at 2% of body mass, consumes about 20% of the whole body energy budget [5]–[8], even though the specific metabolic rate of the human brain is predictably low, given its large size [2]. Moreover, the lower mass-specific brain metabolism in humans is at odds with evidence of up-regulation of genes related to energy metabolism in human evolution [9], [10]. These paradoxes underline our lack of understanding about how brain metabolism scales across brain sizes in evolution. Given that the availability of energy could limit brain size expansion in evolution, particularly in primates [11], the scaling of brain metabolism could influence brain circuitry and activity patterns by exerting selective pressure toward metabolically efficient wiring patterns [12]–[15], neuronal morphology [16] and neural codes [17]–[19].

The declining specific rate of brain metabolism (that is, metabolic rates per gram of tissue) in larger mammalian species, which varies with brain mass raised to an exponent of around −0.14 [2], [20], is usually attributed to a decrease in neuronal density with increasing brain size [21], [2], that is, to an increase in average neuronal size in the tissue [2], or to decreased average firing rates [20], [22]. With fewer, larger neurons per gram of tissue, whether or not accompanied by decreased average firing rates, larger brains would need smaller amounts of energy per gram of tissue to sustain their function. On the other hand, larger neurons are expected to cost more energy, according to an estimate of the distribution of the energy budget among the several energy-consuming processes within a neuron which predicted that, while nearly 80% of a neuron's energy budget go toward glutamate-related neurotransmission, 13% are used to maintain the resting potential of the cell membrane [23]. Considering estimates that neuronal density in the cerebral cortex varies across species with brain size raised to an exponent of −0.3 [21], the slower decrease in specific brain metabolism apparently agrees with an increase in the metabolic cost of larger neurons with increasing brain size with an exponent of the order of 0.15. Despite numerous quantitative studies on the energy requirements of the brain of different species, the metabolic cost per neuron has never been examined, neither in humans nor across species of different brain sizes, although Karbowski [20], based on the supposed scaling of neuronal density with brain size, estimated that cerebral energy per neuron increases with brain size.

However, we have recently shown an increase in average neuronal size in larger brains is not the norm across mammalian species: for instance, while rodent brain structures increase in size gaining neurons whose average size does increase, primate brain structures increase in size through the addition of larger numbers of neurons whose average size remains constant [24]–[26]. As a result, neuronal density decreases with increasing brain size in rodents, but it does not vary consistently with brain size in primates. It is therefore not correct to assume that the declining specific rates of brain metabolism in larger brains result from larger metabolic needs of larger neurons.

To investigate how the metabolic cost of the brain scales with brain size and whether the metabolic cost per neuron increases with neuronal size, it is necessary to examine how total energy consumption relates to the number of neurons in different brains. This analysis was made possible only recently, by the determination of numbers of neurons in the whole brain and its main structures [24]–[28]. The results of this analysis, shown here, suggest that, contrary to expectations, the average metabolic cost per neuron is relatively stable across species, with small variations that are not correlated with neuronal density (and therefore not correlated with neuronal size) nor with brain size. As a result, the total metabolic cost of a brain seems to be a simple, direct function of its number of neurons, each of them constrained to a fixed energy budget per neuron, regardless of brain size.

## Results

Data on the *in vivo* specific utilization rates of glucose (CMRglc) and oxygen (CMRO_2_) by the brain of unanesthetized adult animals are available for six mammalian species [20] for which we have determined total numbers of brain neurons: three rodents (mouse, rat, and squirrel [24], [28]) and three primates (macaque monkey, baboon, and human [25], [27]). Across these species, brain mass varies by 3627-fold, and the number of neurons in the brain varies by 1213-fold (although at different scaling rates across rodents and primates [24], [25]).

**Figure 1:**
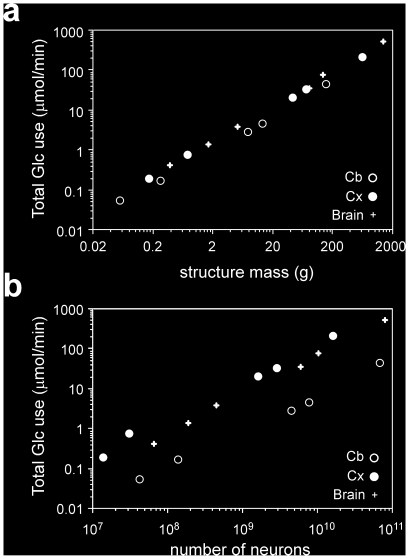
Scaling of total glucose use by the whole brain, cerebral cortex and cerebellum. **a**, total glucose use by the whole brain (+), cerebral cortex (black circles) and cerebellum (white circles) scales with structure mass raised to similar exponents of 0.873, 0.850 and 0.844. **b**, total glucose use by the whole brain (+), cerebral cortex (black circles) and cerebellum (white circles) scales with the number of neurons in each structure in a manner that is best described as a linear function. Whole brain: power exponent 0.988, p<0.0001; linear fit, r^2^ = 1.0, p<0.0001. Cerebral cortex: power exponent 0.944, p = 0.0002; linear fit, r^2^ = 1.0, p<0.0001. Cerebellum: power exponent 0.880, p = 0.0001; linear fit, r^2^ = 1.0, p<0.0001.

Total of glucose and oxygen by the whole brain, cerebral cortex and cerebellum are shown in Tables 1 and 2, calculated as the product of the published specific rates [20] and structure mass (our data). Across the six species, whole brain total glucose use increases with brain mass raised to the power of 0.873 (p<0.0001, 95% CI 0.830–0.915), significantly below linearity, which means that glucose use per gram of tissue decreases with brain mass raised to the power of −0.127, in agreement with the literature [2], [20]. Similarly, whole brain use of oxygen increases with brain mass raised to the power of 0.862 (p = 0.0037, 95% CI 0.635–1.088). In the cerebral cortex and cerebellum, total glucose use also scales with structure mass raised to similar powers of 0.850 and 0.844, respectively (p<0.0001, 95% CI 0.824–0.876 and 0.768–0.919; Figure 1a).

**Table 1:** Glucose consumption averaged per neuron.

Whole brain							
Species	Brain mass*	Glucose use per gram§(µmol/g.min)	Total glucose use (µmol/min)	N_brain_	Glucose use per neuron(µmol/min)	O/N	N/mg
mouse	0.416	0.89	0.370	70.89×10^6^	5.20×10^−9^	0.533	170,408
rat	1.802	0.68	1.225	200.13×10^6^	6.10×10^−9^	0.657	111,060
squirrel	5.548	0.60	3.329	472.44×10^6^	7.05×10^−9^	1.083	85,155
monkey	87.346	0.36	31.444	6.38×10^9^	4.93×10^−9^	1.122	73,043
baboon	148.80	0.44	65.472	10.91×10^9^	6.00×10^−9^	0.828	73,320
human	1508.91	0.31	467.762	86.06×10^9^	5.44×10^−9^	0.983	57,034
variation	3627×	2.9×	1264×	1213×	1.4×	2.1×	3.0×

Cerebral cortex							
Species	Cortical mass*	Glucose use per gram§(µmol/g.min)	Total glucose use (µmol/min)	N_cortex_	Glucose use per neuron(µmol/min)	O/N	N/mg
mouse	0.173	1.10	0.190	13.69×10^6^	1.39×10^−8^	0.881	79133
rat	0.769	0.95	0.730	31.02×10^6^	2.35×10^−8^	1.473	40338
monkey	42.860	0.46	19.716	1.59×10^9^	1.24×10^−8^	2.330	32110
baboon	72.668	0.46	33.427	2.84×10^9^	1.18×10^−8^	1.558	33730
human	632.520	0.34	215.057	16.34×10^9^	1.32×10^−8^	1.363	19540
variation	3656×	3.2×	1132×	1194×	2.0×	2.6×	4.0×

Cerebellum							
Species	Cerebellar mass*	Glucose use per gram§(µmol/g.min)	Total glucose use (µmol/min)	N_cerebellum_	Glucose use per neuron(µmol/min)	O/N	N/mg
mouse	0.056	0.98	0.055	42.22×10^6^	1.30×10^−9^	0.165	753928
rat	0.272	0.62	0.169	137.17×10^6^	1.23×10^−9^	0.211	504301
monkey	7.694	0.37	2.847	4.55×10^9^	0.62×10^−9^	0.204	591390
baboon	13.745	0.32	4.398	7.79×10^9^	0.56×10^−9^	0.067	566752
human	154.02	0.29	44.666	69.03×10^9^	0.65×10^−9^	0.232	448188
variation	2750×	3.4×	812×	1635×	2.3×	1.4×	1.7×

*Our data: references 23–27. Cortical mass refers to both hemispheres, including the hippocampal formation, and excludes subcortical white matter in primates.

§ From [20] (references therein).

**Table 2:** Oxygen consumption averaged per neuron.

Whole brain							
Species	Brain mass*	Oxygen use per gram§(ml/g.min)	Total oxygen use (ml/min)	N_brain_	Oxygen use per neuron(ml/min)	O/N	N/mg
rat	1.802	0.084	0.151	200.13×10^6^	7.54×10^−10^	0.657	111,060
monkey	87.346	0.060	5.241	6.38×10^9^	8.21×10^−10^	1.122	73,043
baboon	148.80	0.034	5.059	10.91×10^9^	4.64×10^−10^	0.828	73,320
human	1508.91	0.035	52.812	86.06×10^9^	6.14×10^−10^	0.983	57,034
variation	837×	2.5×	350×	430×	1.8×	1.7×	1.9×

*Our data: references 23–27.

§ From [20] (references therein).

**Figure 2:**
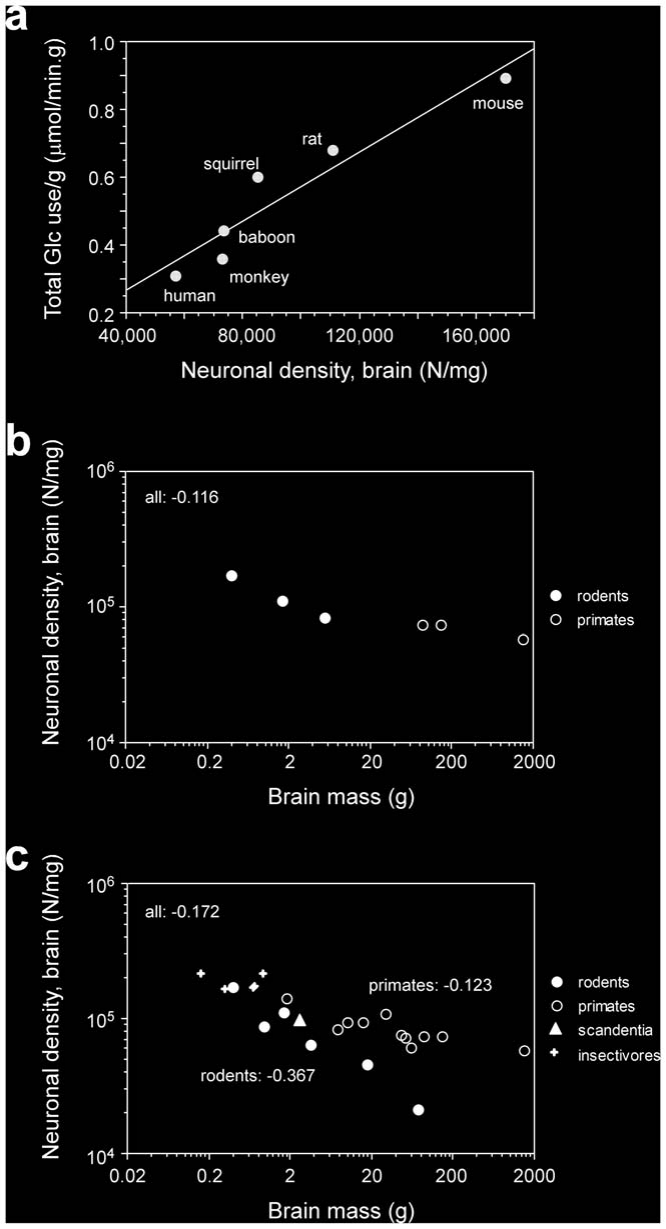
Scaling of average specific glucose use in the brain and neuronal density. **a**, Average glucose use per gram of brain tissue scales linearly with neuronal density. Each point represents the average values for the present species indicated (see Table 1). Average glucose use per gram of brain tissue is best described as a linear function of neuronal density across the species (r2 = 0.906, p = 0.0034), or as a power function of neuronal density with an exponent close to unity (0.986, p = 0.0041). **b**, Neuronal density in the whole brain varies across the six species in the present sample as a power function of brain mass with an exponent of −0.116 (p = 0.0071). **c**, Neuronal density in the whole brain is not a universal function of brain mass: while it does not vary significantly with brain mass across insectivores (crosses), it decreases slightly with brain mass raised to an exponent of −0.123 across primates (p = 0.0016, unfilled symbols); more steeply with brain mass raised to an exponent of −0.367 across rodents (p = 0.0011, filled symbols); and with an intermediate exponent of −0.172 across the ensemble of species (p<0.0001). For subsets of mammalian species, the scaling exponent depends on the particular species.

Remarkably, however, a direct comparison with numbers of neurons shows that total glucose use by the brain as a whole, by the cerebral cortex and also by the cerebellum alone vary with the number of neurons in the structures in a manner that is best described as a linear function across the 6 species (all p<0.0001; Figure 1b), despite the different relationships between structure mass and number of neurons that apply to rodents and to primates [24], [25]. Indeed, the variation in total glucose use by the whole brain or cerebral cortex matches closely the variation in numbers of neurons in these structures across species (Table 1), although not as closely in the cerebellum. Further evidence of the linear scaling of tissue metabolism with its number of neurons is the finding that glucose use per gram of brain tissue increases linearly with neuronal density in the brain (r^2^ = 0.906, p = 0.0034; power exponent, 0.986, p = 0.0041; Figure 2a). The apparent scaling of glucose use per gram of brain tissue with brain size raised to an exponent of −0.127 in the present sample can therefore be explained by a similar apparent scaling of neuronal density in the whole brain with brain size raised to an exponent of −0.116 (Figure 2b). Similarly, the slightly larger exponent of −0.15 that relates specific brain metabolism to brain mass across larger mammalian samples [20] can be accounted for by an apparent scaling of neuronal density with brain mass raised to an exponent that varies depending on the choice of species. Across the mammals that we have examined so far, the apparent exponent for the whole sample is −0.172, close to the exponent of −0.15 for brain metabolism, but notice that there is no universal scaling of neuronal density in the brain with brain mass across all species (Figure 2c). The scaling of brain metabolism, therefore, is best described as a function of the total number of neurons in the brain, regardless of how that relates to brain mass or neuronal density across species.

Consistently with the linear variation in total glucose use depending on the number of neurons in the structure, the estimated average glucose use per neuron within each structure is remarkably constant across species (Table 1), as is the average oxygen use per neuron for the whole brain (Table 2), considering that the small 0.4-fold variations in average glucose use per neuron occur across a 1,000-fold variation in numbers of neurons and total glucose use and a 3-fold variation in neuronal density for the whole brain. Moreover, the small variations in average energy use per neuron do not correlate with structure mass nor with number of neurons in the structure (Spearman correlation, glucose: cerebral cortex, p = 0.2301; cerebellum, p = 0.1615; whole brain, p = 0.8480. Spearman correlation, oxygen: whole brain, p = 0.2987). This indicates that the average energy use per neuron does not scale with number of neurons or brain size. The small variations in the estimated average glucose use per neuron are not correlated with variations in neuronal density across species in any structure (Spearman correlation: cerebral cortex, p = 0.3173; cerebellum, p = 0.6892; whole brain, p = 0.5653), nor with the ratio between non-neuronal and neuronal cells (which approximates the glia/neuron ratio in the tissue; Spearman correlation, cerebral cortex, p = 0.2301; cerebellum, p = 0.5485; whole brain, p = 0.8480). Given that non-neuronal cell density is remarkably constant across these species [24], [25], the inverse of neuronal density can be considered to provide a direct estimate of how average neuronal size varies in the structures. Therefore, the finding that variations in the estimated average glucose use per neuron are not correlated with variations in neuronal density across species suggests that the average energy use per neuron does not scale with average neuronal size (including the soma and all arborizations).

The relatively stable average energy requirement per neuron, whether in the cerebral cortex or cerebellum, allows one to estimate the energy requirement of these structures as a simple linear function of their numbers of neurons (Table 3). Interestingly, the average glucose consumption per neuron is nearly 20× higher in the cerebral cortex (1.50×10^−8^±0.49×10^−8^ µmol glucose/neuron.min) than in the cerebellum (0.87×10^−9^±0.36×10^−9^ µmol glucose/neuron.min; see Table 1 for a comparison within species). Because about 80% of all brain neurons are in the cerebellum, the average glucose use per neuron for the whole brain (5.79×10^−9^±0.76×10^−9^) is lower than the average glucose use by cortical neurons. However, the coordinate scaling of the numbers of neurons in the cerebral cortex and cerebellum, such that the ratio between numbers of cortical and cerebellar neurons remains fairly constant across mammalian species [29], warrants the use of the average glucose consumption per neuron in the whole brain to estimate how the total energy requirement of a mammalian brain depends on the total number of neurons that it contains. As the average brain neuron is estimated to cost 5.79×10^−9^ µmol glucose/min, with no significant difference between rodents and primates, the overall metabolic cost of a brain can be inferred from its number of neurons. Notice that the total glucose use per minute estimated by this method (Table 4) is a very good approximation of the actual measurements made in the available species (Table 1). The similarity between the predicted and measured values validates the calculation of the total energy requirement of a mammalian brain as a linear function of its total number of neurons. Thus, a mammalian brain with 100 million neurons would be predicted to consume 0.579 µmol glucose/min, requiring 0.6 kCal/day; a brain with 1 billion neurons would use 5.79 µmol glucose/min, or 6 kCal/day; and a brain with 100 billion neurons would use 579 µmol glucose/min, or 600 kCal/day, regardless of the volume of these brains (Table 4).

**Table 3:** Estimated cost of mammalian cerebral cortex and cerebellum.

Cerebral cortex				
Number of neurons	Total glucose use per minute (µmol/min)	Total glucose use per day (µmol/day)	Total glucose use per day (g/day)	Total caloric cost per day (kCal/day)
**1 million**	**0.015**	**21.6**	**0.0039**	**0.016**
**10 million**	**0.150**	**216**	**0.039**	**0.155**
Smoky shrew, 10 million^1^	0.150	216	0.039	0.155
Mouse, 13 million^2^	0.195	280.8	0.050	0.202
Rat, 31 million^2^	0.465	669.6	0.120	0.48
**100 million**	**1.500**	**2160**	**0.389**	**1.56**
Agouti, 112 million^2^	1.680	2419	0.435	1.74
Marmoset, 245 million^3^	3.675	5292	0.952	3.81
Capybara, 306 million^2^	4.590	6610	1.190	4.76
Owl monkey, 442 million^3^	6.630	9547	1.718	6.87
**1 billion**	**15.0**	**21600**	**3.89**	**15.55**
Macaque, 1.7 billion^3^	25.5	36720	6.61	26.44
Baboon, 2.9 billion^4^	43.5	62640	11.28	45.10
Orangutan, 5.5 billion^5^	82.5	118800	21.38	85.5
**10 billion**	**150**	**216000**	**38.88**	**155.5**
Human, 16 billion^6^	240	345600	62.21	248.8
**100 billion**	**1500**	**2160000**	**388.8**	**1555.2**

Cerebellum				
Number of neurons	Total glucose use per minute (µmol/min)	Total glucose use per day (µmol/day)	Total glucose use per day (g/day)	Total caloric cost per day (kCal/day)
**1 million**	**0.000873**	**1.257**	**0.00023**	**0.00009**
**10 million**	**0.00873**	**12.57**	**0.0023**	**0.0090**
Smoky shrew, 21 million^1^	0.018	26.40	0.0048	0.0190
Mouse, 42 million^2^	0.037	52.80	0.0095	0.0380
**100 million**	**0.0873**	**125.7**	**0.023**	**0.090**
Rat, 140 million^2^	0.122	176.0	0.032	0.127
Marmoset, 360 million^3^	0.314	452.6	0.081	0.326
Agouti, 680 million^2^	0.594	854.8	0.154	0.615
**1 billion**	**0.873**	**1257.1**	**0.226**	**0.905**
Owl monkey^3^, capybara, 1.1 billion^2^	0.960	1382.8	0.249	0.996
Macaque, 4.6 billion^3^	4.02	5782.8	1.041	4.16
Baboon, 8.0 billion^4^	6.98	10057.0	1.810	7.24
**10 billion**	**8.73**	**12571.2**	**2.26**	**9.05**
Orangutan, 23 billion^5^	20.1	28913.8	5.21	20.8
Human, 69 billion^6^	60.2	86741.3	15.61	62.4
**100 billion**	**87.3**	**125712**	**22.63**	**90.5**

1 From (39);

2 From (23);

3 From (24);

4 From (25);

5 Estimated as 1/3 the number of neurons in the human cerebral cortex;

6 From (26).

**Table 4:** Estimated cost of mammalian brains.

Number of neurons	Total glucose use per minute (µmol/min)	Total glucose use per day (µmol/day)	Total glucose use per day (g/day)	Total caloric cost per day (kCal/day)
**1 million**	**0.00579**	**8.3**	**0.0015**	**0.006**
**10 million**	**0.0579**	**83**	**0.015**	**0.060**
Smoky shrew, 36 million^1^	0.2084	300	0.05	0.2
Mouse, 71 million^2^	0.4111	592	0.11	0.4
**100 million**	**0.579**	**833**	**0.15**	**0.6**
Rat, 200 million^2^	1.158	1667	0.30	1.2
Marmoset, 636 million^3^	3.68	5302	1.0	3.8
Agouti, 795 million^2^	4.60	6628	1.2	4.8
**1 billion**	**5.79**	**8337**	**1.5**	**6.0**
Owl monkey^3^, capybara^2^, 1.5 billion	8.68	12506	2.2	9.0
Macaque, 6.4 billion^3^	37.0	53361	9.6	38
**10 billion**	**57.9**	**83376**	**15**	**60**
Baboon, 11 billion^4^	63.7	91713	16	66
Orangutan, 30 billion^5^	173.7	250128	45.02	180
Human, 86 billion^6^	497.9	717033	129	516
**100 billion**	**579.0**	**833760**	**150**	**600**

1 From (39);

2 From (23);

3 From (24);

4 From (25);

5 Estimated as 1/3 the number of neurons in the human cerebral cortex;

6 From (26).

## Discussion

Although brain tissue is composed of both neuronal and glial cells, the calculation of the average metabolic cost per neuron defined here as the total metabolic cost of a structure divided by its number of neurons is justified by the finding that neurons and astrocytes are metabolically coupled [30]. Neurons and astrocytes use glucose in different manners and quantities: the majority of the glucose used by the brain is taken up by astrocytes and used in anaerobic glycolysis that generates lactate, while the remainder is used by neurons in oxidative glycolysis [31]. However, the glucose taken up by astrocytes is related to neuronal energetics through the stoichiometric coupling to the uptake of glutamate released by synaptic activity and its subsequent conversion to glutamine, while the lactate produced by anaerobic glycolysis is shuttled to neurons and used by them as fuel [32]–[35]. Remarkably, these studies have shown that the same stoichiometric coupling applies to rat [32] and human [33] cerebral cortex *in vivo*. As a consequence, the total glucose use by neurons and astrocytes together is coupled directly to glutamate-mediated synaptic transmission [32], [34], [36], which accounts for 80–90% of total glucose use in the cerebral cortex [32]. Thus, the “average energy cost per neuron” calculated here should be understood as the total amount of glucose-supplied energy that is ultimately required to support one individual neuron, whether it is used indirectly (via astrocytes) or directly by the neurons. Most importantly, the cross-species comparison of the average energy cost per neuron thus defined indicates that this cost does not scale significantly with brain size, neuronal size, or number of neurons. Rather, it suggests that, within structures such as the cerebral cortex and cerebellum, neurons are allotted a fixed energy budget, regardless of their size, across species. Given that the increasing size of a neuronal cell (including all of its arborizations) should impose a higher metabolic demand related to the cost of maintaining membrane polarization [23] and that energy metabolism is coupled to synaptic activity [34], the existence of a fixed energy budget per neuron suggests that neuronal metabolism imposes a series of constraints upon brain structure, function, and evolution, with direct consequences for pathologies in which neuronal metabolism is disturbed, as examined below.

### Brain metabolic scaling with number of neurons, not brain or body size

Studies on the metabolic scaling of the brain usually relate it to the scaling of body size [2], [3], [20], [37]. Such studies have suggested that the steeper increase in brain energy use with brain size (with an exponent of 0.86 [20]) compared to the whole-body energy use with body size (with an exponent of 0.75 [38]) would constitute a metabolic limiting factor in brain expansion, and therefore a reason why brain size usually increases more slowly than body size [37]. The present evidence suggests that metabolic cost is actually an ever more limiting factor to brain expansion that previously suspected, given the steep, linear increase in brain metabolic cost with increasing numbers of neurons.

Karbowski [20] suggests that the similar exponents (of −0.15) of the scaling of the metabolism of the whole brain as well as of several grey matter structures suggests that a common principle might underlie the basal metabolism of different brain structures, such as the presumed homogeneity of synaptic density throughout the grey matter [39], [40], combined with an allometric decrease in neuronal firing rates with increasing brain size [20], [22]. In that case, brain metabolism should be found to scale with brain mass raised to an exponent of 0.85. Alternatively, it has been proposed that the specific metabolism of brain structures is a direct function of neuronal density, such that larger brains, with smaller neuronal densities and larger neurons, have lower specific metabolic rates [20]. Indeed, that author concluded, based on a presumed uniform scaling of neuronal density across mammalian species, that the average energy use per neuron would increase with brain size [20]. However, it has now been demonstrated that there is no uniform scaling of neuronal density with brain size [24], [25], [41], which invalidates the previous estimate: as shown here, the precise exponent that describes the scaling of average neuronal density with brain mass varies across mammalian orders, and, for cross-order comparisons of metabolic scaling such as in [20], is strongly dependent on the choice of species analyzed. Rather, the direct scaling of total brain metabolic cost as a function of the number of brain neurons shown here indicates that the average energy use per neuron is fixed (that is, relatively invariant) across species and orders, such that total brain metabolism is a simple, linear function of the number of neurons that compose that brain. This latter finding can be reconciled with the experimental observation that specific glucose use in the brain decreases with increasing brain mass [20] given the present observation that average neuronal density in the whole brain may, depending on the species analyzed, appears to decrease with increasing brain size at the same rate. Thus, the linear scaling of brain metabolism with its number of neurons also accounts for the larger specific metabolic rates in tissues with larger neuronal densities: the metabolic cost per gram of tissue, as shown here, increases directly with the number of neurons per gram of tissue. Moreover, the fixed energy budget per neuron across brain sizes disputes the traditional view that the ratio between numbers of glial and neuronal cells is related to increased metabolic needs of larger neurons [42]; instead, the glia/neuron ratio may be determined simply by the addition of glial cells of a relatively constant size to neuronal parenchyma consisting of small or large neurons during brain development [24], [43].

In combination with our recent finding that body size is much more variable than the number of neurons in the brain [26], and that different scaling rules apply to the brain of different mammalian orders [24], [25], [41], the scaling of brain metabolism as a function of total numbers of neurons opens the possibility that brain metabolism is not necessarily related to whole body metabolism in any determining way; any apparent relationship might be coincidental, and dependent on the rate with which brain size scales as a function of its number of neurons, which we have shown to vary across mammalian orders [24], [25], [41]. In this way, mammals whose brain scales with economical neuronal scaling rules, such as primates, have a large number of brain neurons for a given body size, and would accordingly be expected to have a larger relative brain metabolic rate than other mammals, such as rodents, which have a smaller number of brain neurons for a same body or brain size [24], [25].

### Metabolic constraints in (human) brain evolution

We have shown previously that the human brain conforms to the neuronal scaling rules that apply to other primates [27], [44]. According to the scaling of brain metabolism with its total number of neurons proposed above, the apparently remarkable use in humans of 20% of the whole body energy budget by a brain that represents only 2% of this mass can be explained quite simply by the large number of neurons in the human brain, about 10× larger than would be expected of a rodent brain of its size [44], given the different neuronal scaling rules that we have found to apply to rodent and primate brains [24], [25].

The finding that total brain metabolism scales linearly with the number of brain neurons implies that in primates, whose brain mass scales linearly with its number of neurons, total brain metabolism scales linearly with brain volume, that is, with an exponent of 1, which is much greater than the much-cited Kleiber's 3/4 exponent that relates body metabolism to body mass [38]. The discrepancy suggests that, per gram, the cost of primate brain tissue scales faster than the cost of non-neuronal bodily tissues, which calls for a modification of the “expensive tissue hypothesis” of brain evolution [11], according to which brain *size* is a limiting factor. In our view, it is not brain size, but rather absolute number of neurons, that imposes a metabolic constraint upon brain scaling in evolution, as individuals with increasing numbers of neurons must be able to sustain their proportionately larger metabolic requirements to keep their brain functional. Animals rely on feeding for their energy intake, which can be very time-consuming. Larger energetic requirements therefore necessitate more time spent foraging [45], and energy intake is further dependent on the availability and quality of foods: orangutans, for instance, spend 4–5 hours per day feeding, but during the months of low fruit availability that is still not enough to provide all the calories required, and the animals lose weight [46]. As illustrated in Table 4, the larger the number of neurons, the higher the total caloric cost of the brain, and therefore the more time required to be spent feeding in order to support the brain alone.

The orangutan brain is about a third the size of the human brain, and therefore, given the linear neuronal scaling rules that apply to primate brains, can be estimated to have roughly 1/3 as many neurons and to require 1/3 as many calories to support the brain alone, that is, about 180 kCal. During the months of low fruit availability, when total caloric intake by females is estimated at about 1800 kCal/day (at best, assuming 100% caloric efficiency of the foods ingested), the orangutan brain is estimated to require about 10% of the total caloric intake, which is less than sufficient to support the body (see above). It can thus be seen how any increase in total numbers of neurons in the evolution of hominins may have taxed survival in a way that may have been limiting, if not prohibitive: a doubling in the number of brain neurons from an orangutan-sized hominin ancestor would have required an additional 180 kCal/day that might not be readily available. In this context, it has been proposed that the advent of the ability to control fire to cook foods, which increases enormously the energetic yield of foods and the speed with which they are consumed [47], may have been a crucial step in allowing the near doubling of numbers of brain neurons that is estimated to have occurred between *Homo erectus* and *Homo sapiens* [48]. The evolution of the human brain, with its high metabolic cost determined by its large number of neurons, may therefore only have been possible due to the use of fire to cook foods, thus enabling individuals to ingest in very little time the entire caloric requirement for the day.

### A fixed energy budget as a constraint for brain function

Increases in neuronal activity, with the associated depolarization and repolarization of cell membranes and cycling of transmitters, are expected to cost more energy [23], [49]. In contrast, in other body organs, such as the liver, the intrinsic metabolic activity of the cells actually *decreases* with increasing body size [50], [51]. The relatively constant values of energy use per neuron across species of smaller neuronal densities (and hence larger neurons) thus suggests that the energy budget per neuron, contrary to the energy budget for other cell types, has been stretched in evolution to remain constant, and therefore might operate close to its limit, imposing a constraint for neuronal activity. This scenario reconciles human brain metabolism with comparative genetic analyses that show that genes related to cell metabolism are among those that exhibit the larger changes in human evolution [9], [10], with evidence of evolutionary pressure for high rates of aerobic energy consumption [52].

Additionally, there is evidence that the energy budget of individual neurons is not only limited across species but also over time, given that it does not accommodate large variations related to neuronal activity. While fractional changes in neuronal firing frequency are directly proportional to changes in energy use [53], the energy demand associated with neuronal activation appears to be small: there is only a 8–12% increase in ATP production with visual stimulation in the awake human visual cortex [54], and a 5% increase in metabolic rate with somatosensory stimulation in the awake somatosensory cortex [55]. Moreover, the approximately 45% reduction in glucose or oxygen consumption with anesthesia-induced loss of consciousness is compatible with the idea that the neuronal energy budget is limiting in the conscious state, such that decreases may compromise function, leading to blackened vision, fainting, and ultimately causing unconsciousness [56]. For the same reason, chronic impairments of neuronal metabolism would be expected to compromise brain function and contribute to brain pathology, which may be the case in epilepsy (due to runaway excitatory activity or directly to metabolic disorders [57]), sepsis [58], mitochondrial disorders, which strongly affect the brain [59], and Alzheimer's disease [60]. Overall, these findings are evidence that the energy budget available for neurons is indeed limiting for the maintenance of healthy brain activity compatible with waking, awareness, and consciousness, and suggest a novel therapeutic approach to some forms of brain disease through the restoration of the energy budget available to neurons.

An energy budget that is relatively invariant across neuronal sizes implies that mechanisms must be in place that adjust firing rate with increasing neuronal size and avoid excessive synaptic activity. Indeed, larger neurons with larger numbers of synapses in culture have recently been found to have sparser connectivity and reduced unitary synapse strength, such that firing rate was preserved across clusters of different sizes [61]. Thus, mechanisms of synaptic homeostasis [62] and plasticity to maintain the number of synapses in check (including the decrease of synaptic markers during sleep [63]), as well as sparse coding, with only a small proportion of neurons firing at high frequencies at any moment [49], [64], [65], may be direct consequences to brain function of a limiting and size-invariant, fixed energy budget per neuron.

## Materials and Methods

This study is entirely based on the analysis of previously published data on brain metabolism and numbers of neurons. Data on the specific *in vivo* utilization rates of glucose (CMRglc) and oxygen (CMRO_2_) by the brain of unanesthetized, resting adult animals were obtained from Karbowski [20], who collected them from various sources. In those studies, the measurements of glucose utilization in all species were performed by [^14^C]2-deoxyglucose uptake [4] in mouse, rat, squirrel and macaque monkey, or by the similar method of [^18^F]fluoro-2-deoxy-D-glucose uptake [66] in baboon, human, and one case of macaque, and are thus directly comparable. Measurements of CMRO_2_ were performed by the Kety-Smith method [67]. Total brain glucose and oxygen utilization rates (Tables 1 and 2) refer to whole brain measurements (Tables S1 and S2 in [20]). Cerebral cortex glucose consumption refers to rates of glucose uptake by the grey matter only, obtained by averaging measurements from six to eight different cortical areas (visual, prefrontal, frontal, sensorimotor, parietal, temporal, cingulate, occipital; Table S11 in [20]). Cerebellar measurements include cerebellar cortex and dentate nucleus, and represent the average of the values listed in Table S14 in [20].

Numbers of neurons that compose the cerebral cortex, cerebellum and whole brain of rodent (mouse, rat, squirrel [24], [28]) and primates (macaque monkey, baboon, and human [25]–[27]) were determined with the isotropic fractionator [68]. Basically, the isotropic fractionator consists of determining the number of cells in a paraformaldehyde-fixed structure after its dissociation in a detergent solution by counting DAPI-stained samples of the resulting isotropic suspension of free nuclei in a hemocytometer under a fluorescence microscope. Numbers of neurons are then determined after establishing the percentage of nuclei in the suspension that are immunoreactive for the universal neuronal marker NeuN [69]. Neuronal densities and non-neuronal/neuronal ratios refer to the ensemble of grey and white matter (which is relatively small) in rodent species, and to the grey matter alone in primate species. Neuronal densities and non-neuronal/neuronal ratios in the cerebellum refer to the entire structure, including the white matter and deep nuclei, in all species.
